# Nephroprotective and Antioxidant Effects of *Jatropha dioica* Extract Against Ischemia–Reperfusion Injury in Wistar Rats

**DOI:** 10.3390/ijms26051838

**Published:** 2025-02-21

**Authors:** Diana Raquel Rodríguez-Rodríguez, Oscar Humberto Mendoza-Hernández, Paula Cordero-Pérez, Verónica Mayela Rivas-Galindo, Diana Patricia Moreno-Peña, Ramiro Tijerina-Márquez, Alondra Michelle Garza-Villarreal, Gabriela Alarcón-Galván, Linda Elsa Muñoz-Espinosa, Homero Arturo Zapata-Chavira, Marco Antonio Hernández-Guedea, Guadalupe Yazmín Solis-Cruz, Liliana Torres-González

**Affiliations:** 1 Liver Unit, Department of Internal Medicine, University Hospital “Dr. José E. González”, Universidad Autónoma de Nuevo León, Monterrey 64460, Nuevo León, Mexico; raquelrodriguez85@gmail.com (D.R.R.-R.); ohmh.oscar@gmail.com (O.H.M.-H.); dpatricia.moreno@gmail.com (D.P.M.-P.); ramiro.tijerina.m@gmail.com (R.T.-M.); alondra.garzavlr@uanl.edu.mx (A.M.G.-V.); linda_uanl@yahoo.com.mx (L.E.M.-E.); 2 Department of Analytical Chemistry, School of Medicine, Universidad Autónoma de Nuevo León, Monterrey 64460, Nuevo León, Mexico; veronica.rivasgl@uanl.edu.mx (V.M.R.-G.); yazmin.sc.qfb@gmail.com (G.Y.S.-C.); 3 Basic Science Department, School of Medicine, Universidad de Monterrey, Monterrey 66238, Nuevo León, Mexico; gabriela.alarcon@patologia.mx; 4 Transplant Service, University Hospital “Dr. José E. González”, Universidad Autónoma de Nuevo León, Monterrey 64460, Nuevo León, Mexico; homero_zapata@yahoo.com (H.A.Z.-C.); marco.hdzguedea@uanl.mx (M.A.H.-G.)

**Keywords:** renal ischemia–reperfusion, *Jatropha dioica*, nephroprotective activity, antioxidant effect

## Abstract

Plant extracts with antioxidant activities have shown nephroprotection against IR injury. *Jatropha dioica* (Jd) possesses antioxidant activity. Our aim was to evaluate the effects of a hydroalcoholic Jd extract against IR injury in Wistar rats. Rats were divided into groups (n = 6): sham (SH); no toxicity (JdTox; 300 mg/kg/day of extract for 7 days); IR (on day 7 [I: 45 min/R: 24 h]); and Jd+IR (same treatment as JdTox; same surgical procedure as IR). AST and LDH were significantly lower in the JdTox. IR exhibited significantly higher CrS, BUN, and MDA compared with SH; Jd+IR showed reductions in these markers. GSH and SOD levels were significantly lower in IR compared with SH; an increase in these markers was observed in the Jd+IR. Histologically, IR showed significant increases in medullary tubular necrosis, medullary protein casts, and medullary vascular congestion compared with SH and JdTox. In Jd+IR, a significant decrease was observed only in medullary tubular necrosis. Therefore, the evaluated hydroalcoholic Jd extract dose showed no nephrotoxicity and hepatotoxicity. Jd extract pretreatment attenuated IR-induced renal injury, as evidenced by the improved serum markers of renal damage and oxidative stress.

## 1. Introduction

Acute kidney injury (AKI) in critically ill patients is an independent risk factor that increases short- and long-term morbidity and mortality, with a tremendous economic impact in terms of health costs. Several investigations have shown that minor increases in serum creatinine (CrS) correlate with a worse prognosis [1]. Numerous risk factors, including drug and toxin exposure, sepsis, and ischemia–reperfusion (IR), frequently contribute to the onset of AKI, leading to a decline in the glomerular filtration rate and acute tubular cell death [2]. In terms of this nephropathy, key biological processes have been identified, including cell death, cellular proliferation, inflammation, fibrosis, and the release of pro-oxidant free radicals, that can exacerbate renal injury and play a crucial role in the development of severe complications [3,4].

IR is a pathological process defined by an initial, transient deprivation of blood supply to a specific organ, culminating in the subsequent re-establishment of perfusion and the simultaneous reoxygenation of tissues. In this final process, paradoxically, a marked exacerbation of tissue damage and the onset of a significant inflammatory cascade occur [5]. IR-elicited tissue injury contributes significantly to morbidity and mortality across a wide range of pathologies, including myocardial infarction, ischemic stroke, acute kidney injury, trauma, circulatory arrest, sickle cell disease, and sleep apnea [6]. IR injury is clinically linked to acute kidney failure secondary to shock or severe hypotension, severe trauma, renal artery thrombosis, renal venous thrombosis, and intra-abdominal hypertension [7]. The consequences of IR injury are particularly critical in medical contexts like surgical procedures and kidney transplantation, where it plays a key role in determining patient outcomes [8].

Given the complex nature of IR injury, various animal models have been developed to understand its impact on kidney function better. Among these, murine experimental models are widely used to investigate the mechanisms that underlie the induction and progression of IR-induced renal damage, as well as to identify potential therapeutic targets to mitigate kidney injury caused by IR events. One such model is IR injury, which induces the necrosis and apoptosis of tubular cells, inflammation, and oxidative stress [2,9,10], ultimately leading to impaired renal function, as assessed by blood urea nitrogen (BUN) and the serum creatinine level (CrS). This IR injury is associated with transiently elevated rates of reactive oxygen species (ROS) production after ischemia, resulting in a complex spectrum of cellular changes, including alterations in mitochondrial function, immune activation, transcriptional reprogramming, microvascular inflammation, endothelial–mesenchymal transition, and cell death [5,11,12,13,14,15,16]. The initial acute response is characterized by the ischemic activation of oxygen-sensitive transcription factors, such as hypoxia-inducible factors, and the activation of the nuclear factor-kappa B signaling pathway [17,18]; this is accompanied by detrimental alteration of antioxidant defense and the significant reduction in the activity of antioxidant enzymes [superoxide dismutase (SOD), reduced glutathione (GSH), catalase, etc.] [19,20].

The use of plants has been shown to attenuate experimentally induced renal IR injury in murine models; this nephroprotective action is mainly attributed to the antioxidant capacity of substances derived from different plant parts (i.e., leaves, flowers, stems, roots, or fruits), which are obtained through various extraction methods [21,22,23,24,25,26,27,28,29].

The genus Jatropha of the family Euphorbiaceae comprises a wide variety of dioecious and monoecious plants, which are primarily native to the Americas, Asia, and Africa, and to which multiple pharmacological properties are attributed. These properties include anti-leishmanial, antimicrobial, antihelminthic, cytotoxic, antiprotozoal, anticoagulant, antispasmodic, antitumoral, antioxidant, analgesic, immunomodulatory, and gastroprotective properties [30,31,32,33].

*Jatropha dioica* (Jd) is a species that is native to Mexico and the southern United States. This heat-tolerant shrub has orange rhizomes, narrow brown-reddish branches, elongated leaves, and sap that turns red when exposed to air, which is why it is traditionally referred to as ‘sangre de drago’ (‘dragon’s blood’) [34,35]. Studies of extracts derived from this plant have reported antioxidant, antibacterial, antiviral, antifungal, hypoglycemic, antidiabetic, cytotoxic, and chemoprotective activities [36,37,38,39,40,41,42,43,44,45,46,47].

Based on the antioxidant and other properties described for this plant, the aim herein was to evaluate a hydroalcoholic extract of Jd as a potential nephroprotective agent against IR injury in Wistar rats.

## 2. Results

### 2.1. Jd Nontoxicity Evaluation

**Figure 1:**
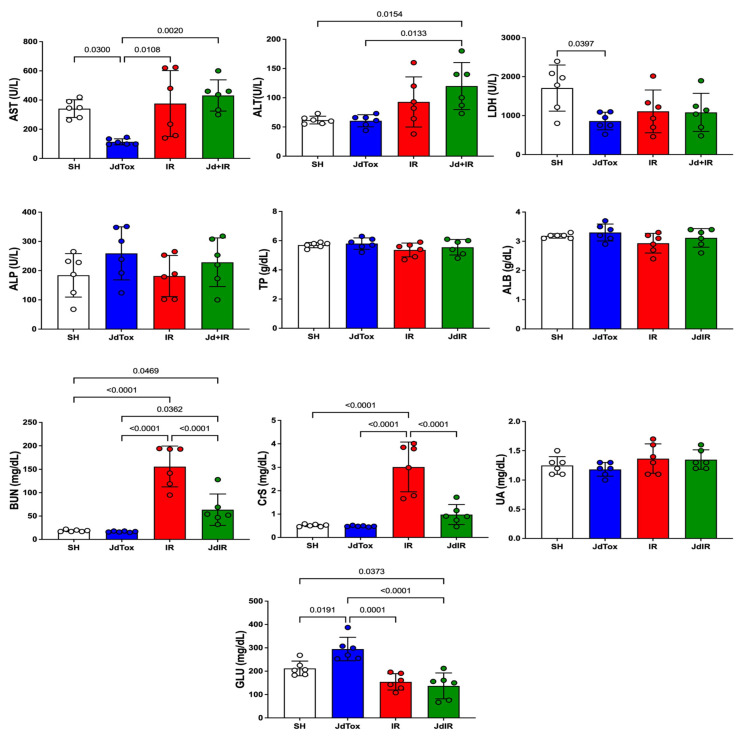
Serum biochemical markers among study groups. AST: aspartate aminotransferase; ALT: alanine aminotransferase; LDH: lactate dehydrogenase; ALP: alkaline phosphatase; TP: total protein; ALB: albumin; BUN: blood urea nitrogen; CrS: creatinine serum level; UA: uric acid; GLU: glucose. Between-group differences were analyzed using one-way ANOVA with Tukey’s post hoc test. Results are expressed as mean ± standard deviation.

**Figure 2:**
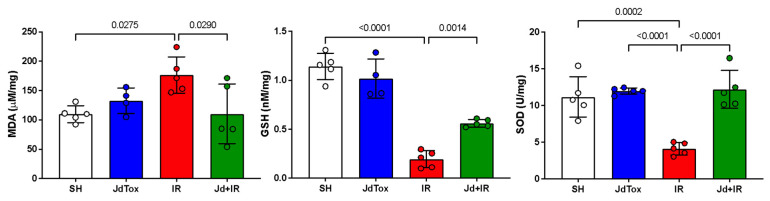
Oxidative stress tissue markers among study groups. MDA: malondialdehyde; GSH: reduced glutathione; SOD: superoxide dismutase. Between-group differences were analyzed using one-way ANOVA followed by Tukey’s post hoc test. Results are expressed as mean ± standard deviation.

Aspartate aminotransferase (AST) and lactate dehydrogenase (LDH) levels were significantly lower in the Jd extract no toxicity group (JdTox) compared with the sham group (SH), with results of 114 ± 21 IU/L vs. 341 ± 61 IU/L (*p* = 0.03) and 861.3 ± 226.4 U/L vs. 1708 ± 591.8 U/L (*p* = 0.03), respectively. Serum glucose (GLU) level in JdTox was significantly higher compared with the level in other groups. There were no significant differences between SH and JdTox in alanine aminotransferase (ALT) (62 ± 7 IU/L vs. 61 ± 10 IU/L), CrS (0.52 ± 0.05 mg/dL vs. 0.48 ± 0.02 mg/dL), BUN (19 ± 2 mg/dL vs. 17 ± 1 mg/dL), albumin (ALB) (3.18 ± 0.08 vs. 3.30 ± 0.29), total protein (TP) (5.7 ± 0.2 vs. 5.8 ± 0.4), alkaline phosphatase (ALP) (184 ± 74 vs. 259 ± 91), uric acid (UA) (1.25 ± 0.15 vs. 1.18 ± 0.12) (Figure 1), malondialdehyde (MDA) (110 ± 14 μM/mg vs. 133 ± 22 μM/mg), GSH (1.1 ± 0.1 nM/mg vs. 1.0 ± 0.2 nM/mg), or SOD (11 ± 3 U/mg vs. 12 ± 1 U/mg) (Figure 2).

### 2.2. Effects of Jd Extract on IR-Induced Renal Damage

CrS and BUN levels were significantly higher in the IR group (IR) compared with the SH group (3.02 ± 1.06 mg/dL vs. 0.52 ± 0.05 mg/dL and 156 ± 44 mg/dL vs. 19 ± 2 mg/dL, respectively; *p* < 0.0001) (Figure 1). Jd extract pretreatment before IR injury (IR vs. Jd with IR group [Jd+IR]) significantly reduced CrS and BUN levels (3.02 ± 1.06 mg/dL vs. 0.98 ± 0.43 mg/dL and 156 ± 44 mg/dL vs. 64 ± 34 mg/dL, respectively; *p* < 0.0001) (Figure 1). There were no significant differences in the other biochemical parameters between IR and Jd+IR.

### 2.3. Effects of Jd Extract on IR-Induced Oxidation

MDA concentration was significantly higher in IR compared with SH (176 ± 31 μM/mg vs. 110 ± 14 μM/mg; *p* = 0.0275). GSH and SOD levels were significantly lower in IR compared with SH (0.19 ± 0.08 nM/mg vs. 1.14 ± 0.13 nM/mg [*p* < 0.0001] and 4 ± 1 U/mg vs. 11 ± 3 U/mg [*p* = 0.0003], respectively) (Figure 2). The MDA concentration in IR was significantly higher than in Jd+IR (176 ± 31 μM/mg vs. 110 ± 51 μM/mg; *p* = 0.0290). GSH and SOD levels were significantly lower in IR compared with Jd+IR (0.56 ± 0.03 nM/mg vs. 19.00 ± 0.08 nM/mg [*p* = 0.0014] and 4 ± 1 U/mg vs. 12 ± 3 U/mg [*p* < 0.0001], respectively) (Figure 2).

### 2.4. Effects of Jd Extract on IR-Histophatological Renal

**Figure 3:**
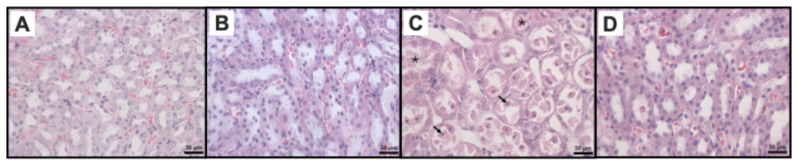
Representative renal micrographs among study groups. H&E staining (400× magnification). (**A**) SH group, (**B**) JdTox group, (**C**) IR group, (**D**) Jd+IR group. The asterisk indicates medullary vascular congestion, while the black arrow indicates medullary tubular necrosis.

The SH group showed no renal cellular structure damage (Figure 3A). The JdTox group exhibited a renal cellular structure like SH (Figure 3B). The IR group showed pathological changes with the medullary watery degeneration or medullary vacuolar degeneration of renal tubular epithelial cells, medullary tubular necrosis, the medullary exfoliation of some renal tubular epithelial cells, medullary protein casts, and medullary vascular congestion (Figure 3C). Renal pathological changes in Jd+IR group were attenuated compared with the IR group (Figure 3D). Histopathological scores are shown in Table 1. A significant increase was observed in the three renal damage histological markers in the IR group compared with the SH group and JdTox group. Pretreatment with the extract before injury induction significantly reduced the medullary tubular necrosis damage only.

## 3. Discussion

In this study, a total hydroalcoholic extract of Jd was evaluated as a potential nephroprotective agent against IR injury in Wistar rats. The results demonstrated that the extract was nontoxic based on hepatic and renal evaluations of various markers. Importantly, it was shown that the extract mitigated renal damage induced by IR, as evidenced by biochemical markers, oxidative stress indicators, and histological findings.

The use of plant extracts as a strategy to attenuate experimentally induced renal IR injury was well documented in previous studies [21,22,23,24,25,26,27,28,29]. Given the wide molecular diversity of plant extracts, it was essential to initially evaluate the nontoxicity of the Jd extract; therefore, we assessed the hepatic and renal effects of the Jd extract using biochemical markers and oxidative stress indicators. It has been reported that increased AST, ALT, and LDH levels indicate the loss of hepatocyte membrane integrity during toxin-induced damage [48]. We observed that a 300 mg/kg dose of Jd extract reduced AST and LDH levels, which could be attributed to compounds previously reported in our studies, such as citlalitrione, 6-epi-riolozatrione, riolozatrione, and jatrophatrione [49], and others like β-sitosterol [50,51], catechin [52], ellagic acid [38,53,54,55], gallic acid [56,57,58], jatrophone A, and jatrophone B [59]; these compounds may have attenuated the damage associated with the surgical procedure, as other authors have reported that this type of compound inhibits the activity of these enzymes [60,61]. Interestingly, ALT levels did not change compared to the SH group, suggesting there was no toxicity at this dose. Other markers, such as TP, ALB, ALP, and UA, showed no changes, which is consistent with previous reports assessing plant extract nontoxicity [62,63].

Renal damage following the metabolism of toxic substances involves multiple mechanisms, evidenced by the increase in CrS, BUN, and MDA levels [64], while SOD and GSH function as potent detoxifiers [65]. In our study, the JdTox group did not show significant changes in CrS, BUN, MDA, SOD, or GSH compared to the SH group, suggesting that the Jd extract does not exhibit renal toxicity. These results are consistent with the reported nontoxicity of Jd in *Artemia salina* lethality bioassays [46,66] and toxicity assays in rats after transplacental exposure [67].

An interesting observation in our study was that GLU levels were significantly higher in the JdTox group compared to other groups. This may be related to the role of peroxisome proliferator-activated receptors (PPARs) in glucose homeostasis [68]. Genetic studies in humans and heterozygous knockout mice have described that reduced PPAR-gamma activity paradoxically improves insulin sensitivity [69]. *Jatropha curcas* has been shown to activate PPAR-gamma in a dose-dependent manner [70]. Furthermore, the use of Jd extract at 34 mg/kg did not reduce glucose tolerance test levels in rats, and at doses of 25, 50, and 100 mg/kg, glucose levels were not reduced in alloxan-induced diabetic rats [45,46]. This may explain the significant increase in glucose observed in the JdTox group. Therefore, it is necessary to evaluate whether Jd extract also acts on PPAR-gamma, as reported with other species of this genus. 

Once it was confirmed that the extract showed no toxicity at the evaluated dose, its effect on the ischemia–reperfusion process was analyzed. Regarding renal function, markers such as CrS and BUN are commonly used to assess the glomerular filtration rate [71]. Previous studies conducted by our group on drugs like misoprostol and plant extracts (e.g., *Juglans mollis*, *Sonchus oleraceus*) demonstrated significant increases in CrS and BUN in renal IR groups compared to sham controls [27,72]. In this study, we observed elevated CrS and BUN levels in the IR group compared to the SH group, and the Jd+IR group exhibited a significant reduction in these markers, suggesting the Jd extract has nephroprotective effects. However, other biochemical parameters evaluated in our study for liver and kidney function, such as ALP, ALB, UA, and TP, did not show significant differences between treatment groups, indicating that liver function was not affected the at the time evaluated. This is consistent with previous reports [73].

Oxidative stress plays a key role in IR injury, as MDA acts as an indirect marker of lipid peroxidation, whereas SOD and GSH serve as markers of antioxidant capacity, playing a role in reversing pathological variations due to oxidative stress damage; thus, they have key functions in the endogenous defense system against ROS [74,75]. As we previously reported in our group’s studies with other treatments in this damage model, we observed an increase in MDA levels and a decrease in SOD levels in renal IR groups compared with sham groups. We also showed how these mediators were modified by drug or extract pretreatment before IR [26,27,72], whereas Karimi G. et al. reported a decrease in GSH in IR compared with sham groups [75]. Here, we also found increased MDA and decreased SOD and GSH levels in the IR group compared to the SH group, while the Jd+IR group showed significant reductions in MDA and increases in SOD and GSH, highlighting the antioxidant potential of the Jd extract.

Histologically, the IR group showed greater renal tissue damage compared with the SH group and was characterized by the presence of protein casts, medullary vascular congestion, and tubular necrosis. These findings are consistent with other studies in which IR-induced renal damage, mainly associated with tubular necrosis, was reported 24 h post-reperfusion [76,77]. Jd administration before IR-induced damage only reduced tubular necrosis, aligning with the reported nephroprotective effects shown by reduced distal and proximal tubules in alloxan-induced diabetic rats [46].

Phytochemical research on Jd has identified secondary metabolites with nephroprotective actions, including β-sitosterol [50,51], catechin [52], ellagic acid [38,53,54,55], and gallic acid [56,57,58]. It has also established the gastroprotective effect of jatrophone A and jatrophone B [59]. Previous studies conducted by our group using HPLC reported that the main composition of the root extract of Jd used in the present study consists primarily of citlalitrione, 6-epi-riolozatrione, riolozatrione, and jatrophatrione [49]. These compounds were previously identified through NMR spectroscopy and X-ray diffraction [41,44]. It is important to mention that the hydroalcoholic extract also contained a variety of glycosidic compounds, which, when analyzed by 13C NMR, showed signals between 60 and 100 ppm, corresponding mainly to carbohydrates. These carbohydrates could not be detected using our validated HPLC method [49]. The antioxidant activity of Jd is largely attributed to polyphenols, which can act as reducing agents, hydrogen donors, and radical scavengers [36,40,78,79]. Specifically, ellagic acid has been shown to protect the kidneys from oxidative stress and mitochondrial dysfunction [38,54,55], while gallic acid significantly improves oxidative stress markers and serum biochemical parameters in renal damage models [56,57,58]. These findings suggest that the nephroprotective effects of Jd observed in this study may be due to the presence of antioxidant compounds. This research emphasizes the biological activity of a total hydroalcoholic extract of Jd, expanding the perspective beyond isolated compounds. The extract’s diverse bioactive constituents, including polyphenols, flavonoids, and terpenoids, may act synergistically, enhancing its therapeutic potential. Furthermore, the use of the total extract, rather than isolated molecules, provides a more practical and resource-efficient approach. This approach favors application in preclinical models and lays the foundation for developing a standardized extract with stringent quality control through chemical fingerprinting.

One limitation of this study was the absence of measurements for additional renal injury markers, such as cystatin C, KIM-1, and NGAL, which could have provided further insight into the extent of renal damage induced by IR. Another important aspect not addressed was the evaluation of the inflammatory signaling pathway associated with the nephroprotective activity of the extract, which limited the understanding of the underlying mechanisms.

## 4. Materials and Methods

### 4.1. Plant and Extraction

Jd var. sessiliflora (Hook) was collected in the municipality of Villaldama, Nuevo León, Mexico, and authenticated at the Institutional Herbarium of the Faculty of Biological Sciences of the Autonomous University of Nuevo León (specimen registration number UAN-24077). Its crude hydroalcoholic extract was obtained according to the method reported by Castro-Ríos et al. [49].

### 4.2. Animals and Ethical Considerations

The experimental procedures were conducted in accordance with the guidelines established by the Official Mexican Standard NOM-062-ZOO-1999 for the production, care, and use of laboratory animals [80]. This project was also approved by the Research Ethics Committee and the Institutional Committee for the Care and Use of Laboratory Animals of the Dr. José Eleuterio González Faculty of Medicine and University Hospital, Autonomous University of Nuevo León (registration number HI22-00002).

Twenty-four Wistar albino rats (both sexes) with an average weight of 265 ± 28 g were used. Rats were kept under standard conditions, being housed in polycarbonate cages in a room at a constant temperature of 24 ± 3 °C on a 12 h light–dark cycle. They had ad libitum access to water and commercial rat pellets (Nutrimix de México, S.A. de C.V., Mexico City, Mexico).

### 4.3. Experimental Study Groups

Rats were randomly distributed (n = 6 per group, 3 females and 3 males) to the following groups:

Sham (SH): vehicle of extract administration (physiological solution with 3% Tween-20) 1 mL/day orally (p.o.) for 7 days, then laparotomy without renal hilum obliteration.

Jd extract no toxicity (JdTox): 300 mg/kg/day extract treatment according to Morales-Velázquez et al. [67] dissolved in 1 mL of the vehicle of extract administration p.o. per day for 7 days, then the same surgical procedure as the SH group.

IR (IR) group: The same treatment as the SH group, then post-administration laparotomy for injury induction via bilateral renal ischemia (45 min) and subsequent reperfusion (24 h).

Jd with IR (Jd+IR) group: The same treatment as the JdTox group, then the same surgical procedure as the IR group.

### 4.4. Laparotomy and IR Injury Induction

In the SH, JdTox, IR, and Jd+IR groups rats were anesthetized using xylazine (Sedaject; Vedilab S.A. de C.V. Reg. SAGARPA Q0088-122; Mexico City, Mexico) via intraperitoneal (i.p.) injection at a dose of 10 mg/kg body weight. Ketamine was used as an analgesic (Anesket; PiSA Agropecuaria, S.A. de C.V. Reg. SAGARPA Q7833-028; Mexico City, Mexico) and administered i.p. at a dose of 100 mg/kg body weight, according to the manufacturer’s specifications.

Following anesthesia, abdominal trichotomy and asepsis were performed using antiseptic solution Microdacyn (Oculus Technologies of Mexico, S.A. de C.V., Guadalajara, Jal., Mexico). This followed by the addition of 20% chlorhexidine gluconate (Farmacéuticos Altamirano de México, S.A. de C.V., Mexico City, Mexico). A midline incision was made to expose the abdominal viscera. In the IR and Jd+IR groups, after dissection, the occlusion of the renal pedicle was performed by placing a vascular clamp (ischemia) bilaterally for 45 min, after which the clamps were removed, initiating the reperfusion time. The acute kidney injury induction was completed with the primary closure of the surgical wound in two layers, using continuous Nylon 3/0 sutures. In the SH and JdTox groups, the same procedure was not performed, as no clamping was applied.

There was a 24 h reperfusion period, during which the rats had free access to food and tramadol solution in water as an analgesic (50 mg/L; Grünenthal GmbH, Stolberg, Germany). At the end of the reperfusion period, rats were administered xylazine and ketamine i.p. at doses of 5 mg/kg and 50 mg/kg, respectively. The surgical wound was reopened, and samples were obtained by exsanguination euthanasia; 5 mL of arterial blood was taken by abdominal aortic puncture, immediately after which both kidneys were removed. The blood sample was centrifuged at 3500 rpm for 15 min to obtain serum for biochemical marker assessments. One half of each kidney was preserved for histological evaluation, while the other half was frozen at −80 °C for subsequent the homogenization and determination of oxidative stress markers malondialdehyde (MDA), superoxide dismutase (SOD), and reduced glutathione (GSH).

### 4.5. Biochemical and Oxidative Stress Marker Analysis

Serum concentrations of CrS, BUN, GLU, UA, ALB, TP, ALT, AST, LDH, and ALP were determined using spectrophotometry (ILab Aries; Instrumentation Laboratory, Milan, Italy) with commercial kits (Instrumentation Laboratory) according to the manufacturer’s specifications.

The degree of lipid peroxidation was quantified by determining the concentration of MDA using the TBARS Assay Kit (Cayman Chemical Company, Ann Arbor, MI, USA). To measure the concentrations, we added 100 μL of tissue homogenate supernatant or standard, 100 μL of sodium dodecyl sulfate, and 4 mL of color reagent to each vial. The vial was heated at 100 °C for 1 h and immediately cooled in an ice bath. It was then centrifuged at 11,000 rpm for 15 min at 4 °C. Subsequently, 150 μL from each vial was transferred to microplate wells. Product absorbance was measured at a wavelength of 540 nm using a microplate reader (Thermo Fisher Scientific Multiskan FC, Carlsbad, CA, USA). Results are expressed as micromoles of MDA formed per liter, normalized to protein concentration using the Bradford method [81].

SOD activity was determined using the SOD Assay Kit (Cayman Chemical Company, Ann Arbor, MI, USA), with a colorimetric assay that uses tetrazolium salt to detect superoxide radicals generated by xanthine oxidase and hypoxanthine. To measure SOD activity, 200 μL of diluted radical detector and 10 μL of tissue homogenate supernatant or standard were added to each well, along with 20 μL of xanthine oxidase. Product absorbance was measured at a wavelength of 460 nm after 20 min using a microplate reader (Thermo Fisher Scientific Multiskan FC). Results are expressed as U/mL, normalized to protein concentration using the Bradford method [81]. One unit (UI) of SOD is defined as the amount of enzyme needed to exhibit 50% dismutation of the superoxide radical.

The GSH concentration was determined using the GSH Assay Kit (Sigma-Aldrich, St. Louis, MO, USA). It was quantified through a kinetic assay in which the catalytic amount (in nanomoles) of GSH continuously reduced 5,52-dithiobis(2-nitrobenzoic acid) to 5-thio-2-nitrobenzoic acid (TNB), and the resulting GSH disulfide was recycled by GSH reductase and NADPH. Subsequently, TNB was measured by spectrophotometry at 412 nm using a microplate reader (Thermo Fisher Scientific Multiskan FC). Results are expressed as nM/L, normalized to the protein concentration using the Bradford method [81].

### 4.6. Histopathological Renal Evaluation

Representative tissue samples were obtained from both kidneys, preserved in 10% phosphate-buffered formalin (pH 7.4), and subsequently embedded in paraffin. Paraffin blocks were sectioned using a microtome at a thickness of 4 nm. After deparaffinization and hydration, sections were stained with hematoxylin and eosin (H&E). Each section was examined under an optical microscope for the histopathological evaluation of renal lesions. Using the indicators proposed in the modified Kobuchi scale [47], a score was assigned for the following: tubular necrosis and protein cylinders (no damage = 0, mild damage = 1, moderate damage = 2, severe damage = 3, very severe damage = 4) and medullary vascular congestion (no congestion = 0, the identification of erythrocytes at 400 magnification = 1, the identification of erythrocytes at 200 = 2, the identification of erythrocytes at 100 = 3, the identification of erythrocytes at 40 = 4).

### 4.7. Statistical Analysis

Data were analyzed using one-way analysis of variance (ANOVA) or the nonparametric Kruskal–Wallis test, alongside Tukey’s or Dunn’s post hoc test, respectively, based on the data distribution, as assessed by the Shapiro–Wilk normality test. Prism software (v. 10.0.0; GraphPad, San Diego, CA, USA) was used for analyses. Data were expressed as mean ± standard deviation or median (interquartile range), as appropriate. *p* < 0.05 was considered statistically significant.

## 5. Conclusions

The Jd hydroalcoholic extract at the dose evaluated herein does not show nephrotoxicity and hepatotoxicity. The fact that Jd extract pretreatment before IR attenuates renal injury was evidenced by the improvement in serum markers of renal damage and oxidative stress. Further studies are needed to evaluate specific, previously reported compounds with antioxidant activities, which may be responsible for the effects observed herein.

## Figures and Tables

**Table 1:** Histological markers of renal damage [47].

Histological Markers of Renal Damage	Study Groups				*p*
	SH	JdTox	IR	Jd+IR	
Medullary tubular necrosis	0 (0–0) ^a^	0 (0–0.25) ^a^	4 (3.75–4)	0 (0–1) ^a^	0.0003
Medullary protein casts	0 (0–0.25) ^a^	0 (0–1) ^a^	4 (3–4)	1.5 (1–2.25)	0.0003
Medullary vascular congestion	0.5 (0–1) ^a^	0.5 (0–1) ^a^	4 (3.75–4)	3 (2–3)	0.0002

^a^ group vs. IR, *p* < 0.05. Between-group differences tested using Kruskal–Wallis with Dunn’s post hoc test. Results are expressed as median (interquartile range).

## Data Availability

The original contributions presented in the study are included in the article; further inquiries can be directed to the corresponding author.
